# Building the Field of Health Policy and Systems Research: Framing the Questions

**DOI:** 10.1371/journal.pmed.1001073

**Published:** 2011-08-16

**Authors:** Kabir Sheikh, Lucy Gilson, Irene Akua Agyepong, Kara Hanson, Freddie Ssengooba, Sara Bennett

**Affiliations:** 1 Public Health Foundation of India, New Delhi, India; 2 School of Public Health and Family Medicine, University of Cape Town, Cape Town, South Africa; 3 Department of Global Health and Development, London School of Hygiene and Tropical Medicine, London, United Kingdom; 4 Ghana Health Service/University of Ghana School of Public Health, Accra, Ghana; 5 School of Public Health, Makerere University, Kampala, Uganda; 6 Health Systems Programme, Johns Hopkins Bloomberg School of Public Health, Baltimore, Maryland, United States of America

## Abstract

In the first of a series of articles addressing the current challenges and opportunities for the development of Health Policy & Systems Research (HPSR), Kabir Sheikh and colleagues lay out the main questions vexing the field.

***PLoS Medicine* Series on HPSR**

Following the *First Global Symposium on Health Systems Research* in Montreux in November 2010, *PLoS Medicine* commissioned three articles on the state-of-the-art in Health Policy and Systems Research (HPSR). Three Policy Forum articles, authored by a diverse group of global health academics, critically examine the current challenges to the field and lay out what is needed to build capacity in HPSR and support local policy development and health systems strengthening, especially in low- and middle-income countries.

*Paper 1*. Kabir Sheikh and colleagues. Building the Field of Health Policy and Systems Research: Framing the Questions.

*Paper 2*. Lucy Gilson and colleagues. Building the Field of Health Policy and Systems Research: Social Science Matters.

*Paper 3*. Sara Bennett and colleagues. Building the Field of Health Policy and Systems Research: An Agenda for Action.

**Summary Points**

- This is the first of a series of three papers addressing the current challenges and opportunities for the development of Health Policy and Systems Research (HPSR). HPSR is a multidisciplinary and interdisciplinary field identified by the topics and scope of questions asked rather than by methodology. The focus of discussion is HPSR in low- and middle-income countries.
- Topics of research in HPSR include international, national, and local health systems and their interconnectivities, and policies made and implemented at all levels of the health system. Research questions in HPSR vary by the level of analysis (macro, meso, and micro) and intent of the question (normative/evaluative or exploratory/explanatory).
- Current heightened attention on HPSR contains significant opportunities, but also threats in the form of certain focus areas and questions being privileged over others; “disciplinary capture” of the field by the dominant health research traditions; and premature and inappropriately narrow definitions.
- We call for greater attention to fundamental, exploratory, and explanatory types of HPSR; to the significance of the field for societal and national development, necessitating HPSR capacity building in low- and middle-income countries; and for greater literacy and application of a wide spectrum of methodologies.

## Introduction

The field of Health Policy and Systems Research (HPSR) is currently experiencing an unprecedented level of interest. The First Global Symposium on Health Systems Research, held in Montreux, Switzerland, in November 2010, is the most recent of a succession of conferences and task force deliberations that have spun off a series of debates about the nature of the field and the future directions it should take. Establishing the identity and terrain of HPSR is part of these debates, which is made difficult by the fact that it is an essentially multidisciplinary field delimited not by methodology but by the topic and scope of research questions asked. In this paper, the first of a series of three addressing the current challenges and opportunities for the development of HPSR, we introduce and map the types of research questions that it has addressed over its natural course of evolution, analyze the nature of current heightened attention, and highlight emerging opportunities and challenges for the development of the field.

We use the extended term Health Policy and Systems Research for a field that is often referred to simply as Health Systems Research. For us, the broader term better captures the terrain of work it encompasses because it explicitly identifies the interconnections between policy and systems, and highlights the social and political nature of the field. The geographical focus of our concern is low- and middle-income countries (LMICs) [1], but we suggest that our approach also has value for high-income countries. Our understanding of the evolution of HPSR draws primarily from the English language literature, which we acknowledge as a limitation. However, this reflects global discussion about the field, which has tended to neglect literature in other languages.

## Evolution of a Question-Driven Field

Compared to other health research traditions, HPSR has a short but eclectic history. Many of the researchers who have led its development have brought social science perspectives, including health economics, sociology, political science, and anthropology, complementing the contributions of individuals and institutions engaged in delivering health services. A rearview look at these diverse antecedents reveals that HPSR has taken form from, and continues to be shaped by, questions bubbling up from the field—whether those asked by curious social scientists and observers drawn to the complexity of health systems and seeking to support change within them, or by public health specialists and health systems actors impelled to resolve practical concerns of service delivery. The state of HPSR in terms of methodological sophistication and advances results both from the independent contributions of discrete traditions of enquiry, as well as from the mixing of disciplinary influences—it is simultaneously, therefore, a multidisciplinary and interdisciplinary field.

### Focus Areas in HPSR

**Figure 1:**
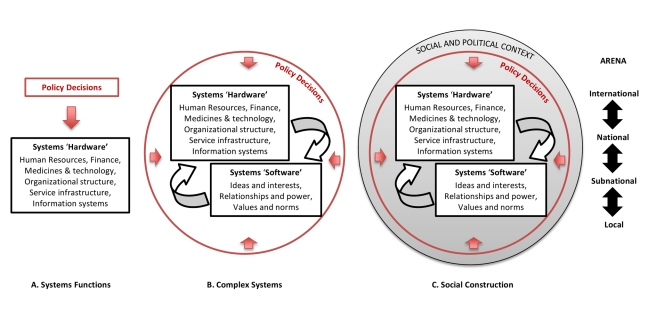
Health policy and systems: alternative perspectives.

Figure 1 illustrates how understanding of subjects of inquiry in HPSR varies depending on the perspective taken [2]. Health policy is commonly seen as the formal written documents, rules, and guidelines that present policy makers' decisions about what actions are deemed legitimate and necessary to strengthen the health system and improve health. Increasingly, however, it has been understood to encompass, importantly, the processes of decision-making at all levels of the health system and the wider influences that underpin the prioritisation of policy issues, the formulation of policy, the processes of bringing them alive in practice, and their evaluation [3].

Definitions of health systems, meanwhile, have been based mainly on their utility in the achievement of health outcomes. The World Health Organization (WHO) building blocks approach is one such popular classification, which conceptualizes health systems in the functional or instrumental terms of its constituent “hardware”—finance, medical products, information systems, levels and types of human resources, forms of service delivery, and governance understood as organizational structures and legislation, for example. [4]. It also recognises that the system encompasses both the suppliers of policy, services, and interventions, and the communities and households intended to benefit from them who, as citizens, also play important roles in policy change. However, in addition to these concrete and tangible expressions of health systems, the “software”—by which we mean the ideas and interests, values and norms, and affinities and power that guide actions and underpin the relationships among system actors and elements—are also critical to overall health systems performance. Alternative formulations of complex health systems have been influenced by economic theories of markets and political institutionalism, drawing attention to non-linear and dynamic relationships between different parts of health systems, and to the role of software and its interplay with the visible and quantifiable hardware of systems [5],[6]. Finally, the influence of discursive and critical theory, through contributions from policy analysis and sociology, have brought an emerging recognition that health systems and policies are artifices of human creation, embedded in social and political reality and shaped by particular, culturally determined ways of framing problems and solutions [7],[8]. Acknowledgement of these influences was another reason for our choice of field name, and has radical implications for research, and also for how we envision change in systems.

International, national, subnational (provincial), and local arenas, as well as their respective intersections, are each equally part of this broader conception of the constructed reality of health systems, with the local arena encompassing not only delivery of services, but also the worlds of health providers; activities of provision, protection, and promotion of health in local communities and households; and systems of local health governance.

### A Typology of Questions

The range of questions encompassed by HPSR is broad. In the first place, there are different levels of analysis—macro-level analysis analyzes the architecture and oversight of systems, meso-level analysis focuses on the functioning of organizations and systemic interventions, and micro-level analysis considers the roles of individuals involved in activities of health provision, utilization, and governance, and how systems respectively shape and are shaped by their decisions and behaviour. Research questions can also be classified by their *intent*, which may broadly be seen to be either 1) normative/evaluative or 2) exploratory/explanatory in nature [9]. Table 1 maps types of questions, with indicative examples, according to the level and intent of analysis—this may be seen as a step towards constructing a broad church (or mosque, or temple) for HPSR.

**Table 1:** Examples of HPSR questions by level of analysis and type of question.

*Level of analysis:*	MACROArchitecture and Oversight of Systems	MESOFunctioning of Organizations and Interventions	MICROThe Individual in the System
*Intent of question:*			
Normative/Evaluative	→ How can political parties be effectively involved in a country's health planning process for universal health coverage?→ Does a new financing mechanism protect the poorest households from the catastrophic costs of accessing care?→ Can community accountability mechanisms have impact on health outcomes?	→ How can access to and uptake of a screening and treatment programme for an epidemic condition be maximised?→ What are the reasons for low efficiency of community governance structures in administering a decentralised fund scheme?	→ What financial and non-financial incentives will best encourage health workers to locate in underserved communities?→ Does individual coaching offer better support to health system managers than formal training?→ Do conditional cash transfers encourage individual behaviour change in use of health care?
Exploratory/Explanatory	→ Why do informal health markets continue to flourish in areas where publicly provided services are adequate?→ What norms underpin the effective exercise of oversight by communities?	→ How do pay-for-performance arrangements interact with local accountability structures?→ Why do organizations involved in the implementation of health policies prioritize some aspects of their mandate more than others?→ How has the introduction of subsidies for institutional deliveries changed household birthing practices?	→ Why do frontline health providers frequently diverge from recommended clinical guidelines?→ How has engaging traditional practitioners in government clinics changed laypersons' perceptions of public services?

## The New Interest in HPSR

The recent upsurge of interest in HPSR, whilst partly a culmination of the efforts of earlier generations of researchers, owes much to recognition of its importance for the success of health interventions and programmes, and the changing macroeconomic environment of international health. As funding for health scaled up during the period 2000–2008, it became evident that Millennium Development Goal targets would not be achieved due to weak health systems. This catalyzed interest in the health systems field by international alliances and donors, as well as a nascent advocacy movement, partly synergistic also with HIV/AIDS advocacy. Specific departments within international organizations, such as the Health Systems and Services cluster at WHO, were established and new research organizations focusing on health systems research, such as the Alliance for Health Policy and Systems Research and the Institute for Health Metrics and Evaluation, emerged. During the past decade a series of conferences and task forces on health research, including the International Conference on Health Research for Development, Bangkok, 2000, and ministerial meetings in Mexico City in 2004 and Bamako in 2008, had a strong focus on practical, operational questions, and this was frequently framed as health systems research. In addition to these global trends, innovative health reforms in emerging economies such as Brazil, China, India, and Thailand have created enthusiasm around the scope for system-level interventions.

The upsurge in interest is a reflection of the wide-ranging relevance of HPSR, as well as a commentary on the overdue need for the elevation of this research field to the stature of the dominant traditions of health research. There are numerous potential benefits of the current concern, for the future of the field:

- New insights into key problems and focus areas, particularly resulting from the participation of actors representing the clinical and epidemiological sciences, and from reflection on operational and service delivery experiences.
- Opportunities for development of a range of new research methodologies drawing from diverse disciplinary perspectives.
- Expansion of funding platforms and increased funding for HPSR in LMIC contexts.

However, the combination of heightened attention in a short span of time with the differing interests of involved actors has altered how HPSR is perceived and framed in the present day. In itself this presents significant threats for the balanced and holistic development of the field—three of the most important threats are discussed here.

### Skewed Balance in Focus Areas and Questions

HPSR has previously played an important role in exploring the societal relevance and purpose of systems and interventions, and helping shape systems values [10],[11]. Another important potential function of HPSR is to examine software elements such as power and trust that have been demonstrated to be key determinants of health systems performance, and success of health policies [12],[13]. However, the current focus of the field of research is frequently framed around the hardware of health systems, and less around its software (See Figure 1). This is underpinned by the dominance of the positivist paradigm of knowledge which, with its claims to value-neutrality, has led to health systems being seen primarily as vehicles for technological solutions rather than being grounded in political and social contexts with underlying power structures, interests, and interdependencies.

Secondly, particular arenas of health policy and systems remain poorly addressed. The current framing of HPSR has tended to foreground issues around the delivery of specific interventions and services (often specific programmes of disease control, and often driven by global actors and agendas) rather than the existing national and sub-national systems and institutions through which they are administered. The influence of local political cultures and practices over system performance is another critical area of neglect in HPSR—yet organizational ethos and inter-organizational relationships are key determinants of how and whether policies get implemented.

Finally, the current framing of HPSR has broadly been skewed towards short-term pragmatic and operational questions, rather than being oriented towards theoretical development. Within the predominantly normative/evaluative focus of current questions there has also been a particular emphasis on deriving generalizable solutions that can be applied internationally, rather than working towards resolving specific societal problems through engagement with national and subnational policy planners. The dominant trend of donor-driven HPSR with an emphasis on addressing operational needs could have the effect of undermining the capabilities of research and academic organizations to address more fundamental, exploratory, and explanatory questions around the character and relevance of health policies and systems in real-world social and political contexts [14].

### “Disciplinary Capture”

The stakeholders—including researchers, funders, and journal editors—who are converging on HPSR, come from diverse disciplinary backgrounds. In this plural field, probably the most significant risk to its development lies in the lack of mutual understanding and respect across the range of contributory disciplines. In HPSR, as in health research in general, the dominant group of actors (in terms of both volume and influence) are those involved in the delivery of health services (primarily medical professionals). These actors work mainly in the frame of the dominant health research traditions—including epidemiological, biomedical, and clinical research—and commonly employ a positivist paradigm of knowledge [15].

Disciplinary capture may occur if this knowledge frame, with its attendant criteria of research quality, is superimposed on the entire field for want of a wider understanding of alternative paradigms of knowledge. The quantitative methods and measures commonly used in these dominant health research fields may be over-utilised in the service of HPSR questions, where qualitative, inductive, or participatory methods may work better. Frequently too, the rigour of HPSR is assessed with inappropriate standards, extrapolated from the dominant research traditions [15].

### Inappropriate Definitions

As HPSR is beginning to take shape from its multitude of influences, there is an undoubted and widely acknowledged need to enhance clarity and consensus on research methods, and deepen the theoretical foundations of the field. Prevailing attempts to characterize the field have broadly focused on offering definitions of HPSR, sometimes seeking to distinguish it from related areas of research [16]. It is argued that these definitional attempts have utility in guiding the allocation of global funds. However, for much the same reason, they risk constraining the understanding and natural development of the field, and may lead to the neglect and “crowding out” of particular types of HPSR that do not fit neatly into popular definitions, such as the examples of overlooked focus areas and types of questions cited above. In addition to militating against the hitherto inclusive, question-driven ethos of the field, such territorial approaches also present significant problems when definitions are inappropriately narrow or incomplete (Box 1).

**Box 1.** Narrow Definitions: Two Case Studies.

1. *Implementation Research:* In the world of public policy analysis, research on implementation is a far-reaching terrain of work synonymous with the study of governance, clearly a central element of HPSR. Implementation research in this understanding is a four-decade-old field built on a wide foundation of empirical and theoretical work, propelled by vibrant debates between top-down and action-centered (or bottom-up) thinking. While top-down approaches analyze the ineffectiveness of public policies at all levels [22], and aim to diagnose and resolve implementation deficits, action-centred theorists see implementation as a relationship between policy and action, involving negotiations and interactions in social and political contexts, and use social science research methods to understand “what actually happens, how and why” [23]. However, current definitions of Implementation Research (IR) in recent influential articles appear to overlook this entire paradigm and the extensive body of research within it [16]. IR in this interpretation focuses on the concerns of programme managers regarding the effectiveness of specific health interventions. In restricting IR to the objective of facilitating predetermined programmatic solutions, a broad terrain of understanding and research is effectively reduced to a topic area with a predominant top-down focus. The narrow enunciation of delivery of health interventions or programmes also excludes an understanding of implementation of other levels (e.g., global, sectoral, institutional) and domains of policy (e.g., health workforce, regulation, financing), each a significant area of research enquiry.
2. *Impact Evaluation:* A related movement is the current ascendance of the field of “impact evaluation”, with its emphasis on a narrow range of “robust” methods that are believed to ensure an unbiased measure of intervention impact. This restriction on admissible study designs is also seen in Cochrane reviews of health system interventions undertaken through the Effective Practice and Organisation of Care (EPOC) group, which also holds the randomised design as the “gold standard”. Yet, such methods are often ill-suited to the evaluation of complex interventions (which would include many, if not all, health system strengthening interventions) where the causal mechanism is multifaceted and contextual factors play an important role. For instance, when there is a change in policy at the national level, there may be no obvious group against which change can be assessed, nor the opportunity to randomize units to intervention or control group. Even where it is possible to introduce variation in policy at the local level, reliance on randomised methods to rule out confounders in the measurement of impact may lead to a neglect of understanding of the specific elements of the context that are responsible for programme success or failure. For these interventions, it would seem wise to admit a wider variety of study designs for examining and interpreting programme impact, and for generating knowledge that can be generalised to other contexts.

Furthermore, LMIC health systems are also changing rapidly, and moves to delimit the field with narrow definitions may well be short-sighted. Emerging phenomena such as the changing roles of health care professionals, increasing health literacy, commercialization of health, and technological innovation—for information and communication, diagnosis, and treatment—will each pose new questions, necessitating a relatively open-ended outlook on the topics and approaches of enquiry constituting HPSR [17]–[20].

## Framing HPSR: A Balanced Agenda

HPSR owes much of its present-day prominence to its utility in supporting the effective implementation of health interventions and programmes. The key underlying assumption in this popular use of HPSR is that scientific-technical solutions for health concerns have previously been proven through epidemiological, biomedical, or clinical research, and the problem lies in actualization due to deficiencies in how the solution is administered by the health system, and necessitating enquiry into system “bottlenecks”. Consequently, in the broader schema of health research, research questions pertaining to health policy and systems have tended to occupy the position of being secondary or subsequent to the primary scientific-technical question.

It is important to recognize that HPSR does not exist only for reasons of its usefulness in addressing the constraints of specific health interventions, nor does it need to mimic the systems of knowledge generation prevalent in the dominant health research traditions. HPSR may logically be conceptualised in a complementary and *equivalent*, not subordinate, position to the other health research traditions in the quest for solutions to health concerns. It is a free-standing field of research with diverse, serious goals including supporting societal development and self-sufficiency of nations and communities in the long term, and examining the appropriateness of scientific-technical solutions when applied in real-world contexts [21].

HPSR should have room for multiple foci of enquiry and types of research questions, and a wide spectrum of methodological approaches. The normative and evaluative functions of HPSR are well established, but there is also scope in HPSR for more fundamental, exploratory, and explanatory questions. Acceptance of and support for fundamental research is an important signifier of the maturation and wholeness of a field. Fundamental research has instrumental value in aiding health systems performance, and also serves long-term developmental goals. It is essential in shaping policy, and is the basis for a body of reference knowledge and a firm theoretical platform—baselines on which future researchers can build.

While the awakening of interest in HPSR contains great opportunities, we are also concerned that the disciplinary biases, premature enunciation of definitions, and the skewed balance of questions currently prioritised within HPSR weakens rather than strengthens the field, and so could undermine its potential to facilitate long-term goals of societal development. The practical challenges ahead, particularly as we seek to build capacity for HPSR in LMICs, include balanced growth and promoting wider literacy of the inherent diversity and varied potentialities of the field. These questions are addressed in the two forthcoming papers in this series.

## Abbreviations

HPSR: Health Policy and Systems Research
LMIC: low- and middle-income country
